# Group B streptococcus vaccination in pregnant women with or without HIV in Africa: a non-randomised phase 2, open-label, multicentre trial

**DOI:** 10.1016/S1473-3099(15)00484-3

**Published:** 2016-05

**Authors:** Robert S Heyderman, Shabir A Madhi, Neil French, Clare Cutland, Bagrey Ngwira, Doris Kayambo, Robert Mboizi, Anthonet Koen, Lisa Jose, Morounfolu Olugbosi, Frederik Wittke, Karen Slobod, Peter M Dull

**Affiliations:** a Malawi Liverpool Wellcome Trust Clinical Research Programme, University of Malawi College of Medicine, Blantyre, Malawi; b Division of Infection and Immunity, University College London, London, UK; c Medical Research Council: Respiratory and Meningeal Pathogens Research Unit, University of the Witwatersrand, Johannesburg, South Africa; d Department of Science and Technology/National Research Foundation: Vaccine Preventable Diseases, University of the Witwatersrand, Johannesburg, South Africa; e National Institute for Communicable Diseases: a division of National Health Laboratory Service, Centre for Vaccines and Immunology, Johannesburg, South Africa; f Institute of Infection and Global Health, University of Liverpool, Liverpool, UK; g GlaxoSmithKline, Cambridge, MA, USA; h Novartis Vaccines and Diagnostic, Siena, Italy

## Abstract

**Background:**

Neonates born to women infected with HIV are at increased risk for invasive group B streptococcus (GBS) disease. We aimed to compare safety and immunogenicity of trivalent glycoconjugate GBS vaccine in pregnant women with and without HIV in Malawi and South Africa.

**Methods:**

In our non-randomised phase 2, open-label, multicentre study, we recruited pregnant women attending two antenatal clinics, one in Blantyre, Malawi, and one in Soweto, Johannesburg, South Africa. Participants were divided into three groups on the basis of their HIV infection status (no infection, infection and high CD4 cell count [>350 cells per μL], and infection and low CD4 cell count [>50 to ≤350 cells per μL]) and received a 5 μg dose of glycoconjugate GBS vaccine (serotypes Ia, Ib, and III, with CRM_197_ [Novartis Vaccines, Siena, Italy]) intramuscularly at 24–35 weeks' gestation. GBS serotype-specific antibody concentrations were measured before vaccination (day 1), day 15, day 31, and at delivery, and in infants at birth and day 42 of life. The primary outcomes were safety in mothers and infants and the amount of placental transfer of GBS serotype-specific antibodies from mothers to their infants. All immunogenicity and safety analyses were done on the full analysis set, including participants who, or whose mother, correctly received the vaccine and who provided at least one valid assessable serum sample. This study is registered with ClinicalTrials.gov, number NCT01412801.

**Findings:**

270 women and 266 infants were enrolled between Sept 26, 2011, and Dec 4, 2012 (90 women and 87 infants without HIV, 89 and 88 with HIV and high CD4 cell counts, and 91 and 91 with HIV and low CD4 cell counts, respectively). Seven women were lost to follow-up, six withdrew consent, one died, and two relocated. Eight infants died or were stillborn and two were lost to follow-up. Across serotypes, fold change in antibody concentrations were higher for the HIV-uninfected group than the HIV-infected groups. Transfer ratios were similar across all three groups (0·49–0·72; transfer ratio is infant geometric mean antibody concentration in blood collected within 72 h of birth divided by maternal geometric mean antibody concentration in blood collected at delivery); however, at birth, maternally derived serotype-specific antibody concentrations were lower for infants born to women infected with HIV (0·52–1·62 μg/mL) than for those born to women not infected with HIV (2·67–3·91 μg/mL). 151 (57%) of 265 women reported at least one solicited adverse reaction: 39 (45%) of 87 women with HIV and low CD4 cell counts, 52 (59%) of 88 women with HIV and high CD4 cell counts, and 60 (67%) of 90 women in the HIV-uninfected group. 49 (18%) of 269 women had at least one adverse event deemed possibly related to the vaccine (six [7%] in the HIV and low CD4 cell count group, 12 [13%] in the HIV and high CD4 cell count group, and 21 [23%] in the HIV-uninfected group), as did three (1%) of 266 neonates (zero, two [1%], and one [1%]); none of these events was regarded as serious.

**Interpretation:**

The vaccine was less immunogenic in women infected with HIV than it was in those not infected, irrespective of CD4 cell count, resulting in lower levels of serotype-specific maternal antibody transferred to infants, which could reduce vaccine protection against invasive GBS disease. A validated assay and correlate of protection is needed to understand the potential protective value of this vaccine.

**Funding:**

Novartis Vaccines and Diagnostics division (now part of the GlaxoSmithKline group of companies), Wellcome Trust UK, Medical Research Council: Respiratory and Meningeal Pathogens Research Unit.

## Introduction

Several African countries including Malawi, Liberia, and Ethiopia have met their Millennium Development Goals for child mortality reduction; however, neonatal deaths caused by infections, preterm birth, and birth asphyxia account for 44% of mortality for children younger than 5 years.^1^ Group B streptococcus (GBS) has been identified as a leading cause of neonatal sepsis and meningitis in several countries across sub-Saharan Africa,^2^ and is therefore a crucial target for public health intervention. In Africa, the reported incidence of early-onset invasive GBS disease varies across studies from 0 to 2·1 per 1000 livebirths, whereas late-onset invasive GBS disease varies from 0 to 0·89 per 1000 livebirths, with case fatality rates ranging from 13% to 46%.^2^ Intrapartum antibiotic prophylaxis has substantially reduced, although not eliminated, early-onset invasive GBS disease in high-income countries.^3^ Intrapartum antibiotic prophylaxis is difficult to implement in resource-poor settings and has little effect on the incidence of late-onset invasive GBS disease.^4^

**Research in context**

**Evidence before this study**

We searched PubMed and Web of Science for studies on group B streptococcus (GBS) vaccines published before Dec 1, 2015, using the search terms “Group B Streptococcus vaccine” and combinations thereof. We did not find any previous GBS vaccines that had been tested on pregnant women infected with HIV, and no published research on CRM_197_-conjugated GBS vaccines in human beings.

**Added value of this study**

This is the first study investigating the safety and immunogenicity of a candidate glycoconjugate GBS vaccine in pregnant women infected with HIV and their infants, and shows that the vaccine is both immunogenic and has a good safety profile, although it was less immunogenic in women infected with HIV. The study also adds to previous evidence of the safety and immunogenicity of GBS vaccines in healthy pregnant and non-pregnant women, in earlier clinical trials. This is the first GBS vaccine immunogenicity study to be done on the African continent that has included participants from outside South Africa.

**Implications of all the available evidence**

Given that GBS is a leading cause of neonatal sepsis and meningitis across sub-Saharan Africa, this vaccine offers a potential pathway to reduce infection-related neonatal death and disability in high-burden settings. However, since the vaccine was less immunogenic in women infected with HIV than in those not infected, irrespective of CD4 cell count, validated correlates of protection need to be identified to improve understanding of the potential protective value of this GBS vaccine for pregnant women and their infants, particularly in high HIV seroprevalence countries.

Glycoconjugate vaccines against other capsulated bacteria, such as *Neisseria meningitidis, Streptococcus pneumoniae,* and *Haemophilus influenzae* type b, have proved highly effective in many settings.^5^, ^6^, ^7^ Because neonatal GBS disease develops rapidly after birth, administration of a glycoconjugate GBS vaccine to infants is unlikely to prevent disease and therefore maternal immunisation during pregnancy has been identified as the best strategy to prevent both early-onset and late-onset invasive GBS disease, and therefore possibly to reduce the incidence of stillbirth and miscarriage.^8^ Maternal immunisation against other diseases, such as tetanus, has proved highly effective and acceptable in many low-income and middle-income countries.^9^, ^10^

About 5·3% of pregnant women in sub-Saharan Africa are thought to be infected with HIV, and although efforts to prevent mother-to-child transmission across the continent are having an effect, rates of neonatal exposure to HIV remain high.^11^ Neonates born to women infected with HIV are at increased risk for invasive GBS disease in high-income and middle-income countries.^12^, ^13^ The risk for late-onset invasive GBS disease (occurring in neonates aged between 7 and 90 days) is 3·18 to 19 times greater in neonates born to mothers infected with HIV than in those born to mothers not infected with HIV, and the risk for early-onset GBS disease (occurring in neonates aged <7 days) is 1·7 times greater.^13^, ^14^, ^15^ A study^11^ in Belgium reported that 1·55% of infants born to mothers infected with HIV developed invasive GBS disease, compared with 0·08% of infants born to mothers not infected with HIV over the same time period. Similarly, a study^15^ in South Africa reported the incidence of invasive GBS disease to be higher in infants exposed to HIV (4·46 per 1000 livebirths) compared with infants not exposed to HIV (1·98 per 1000 livebirths). The increased susceptibility to invasive GBS disease in neonates born to women infected with HIV, despite most of these babies not being infected with HIV, is probably due to lower concentrations of naturally acquired serotype-specific capsular antibodies and reduced transplacental transfer in mother–newborn dyads in which the mother is infected with HIV than in dyads in which the mother is not infected.^15^ Response to vaccination is known to be impaired in individuals with HIV infection.^16^ A previous trial^17^ of three doses (0·5 μg, 2·5 μg, and 5·0 μg) of a glycoconjugate GBS trivalent vaccine in women not infected with HIV in South Africa showed that the vaccine was well tolerated and immunogenic.

In the present study, we explore the hypothesis that HIV infection affects the safety and immunogenicity of the 5·0 μg formulation of GBS vaccine in pregnant women and their infants in Malawi and South Africa. We aimed to assess placental transfer of GBS serotype-specific antibodies in patients with and without HIV, as well as post-vaccination concentrations in mothers and safety.

## Methods

### Study design

This non-randomised phase 2, open-label, multicentre study was done in two antenatal clinics, one in Blantyre, Malawi, and one in Soweto, Johannesburg, South Africa, between September, 2011, and December, 2012. Pregnant women were enrolled sequentially by study nurses into three groups in a 1:1:1 ratio on the basis of their HIV infection status and CD4 cell count until each of the groups was filled; the groups were HIV uninfected, HIV infected with a high CD4 cell count (>350 cells per μL), and HIV infected with a low CD4 cell count (>50 to ≤350 cells per μL).

The study was done in accordance with the Declaration of Helsinki and the International Council for Harmonisation of Technical Requirements for Pharmaceuticals for Human Use Guidelines for Good Clinical Practice. Written informed consent was obtained from women before enrolment. The protocol was approved by the National Health Sciences Research Committee (Malawi) and the University of Witwatersrand, Human Research Ethics Committee (South Africa). Use of an investigational GBS vaccine was approved by the Pharmacy, Medicines & Poisons Board, Malawi, and the Medicine Control Council, South Africa.

### Participants

Pregnant women aged 18–40 years between 24 and 35 weeks' gestation were eligible. All women attending the clinics at the study sites were informed of the study. Those who fulfilled the study eligibility criteria and agreed to take part were enrolled after giving informed written consent. Women infected with HIV were eligible if their CD4 cell count was more than 50 cells per μL and they had WHO stage I or II disease. Exclusion criteria included receipt of a vaccine 15 days before enrolment (except tetanus toxoid and non-alum adjuvanted influenza vaccines); severe allergic reaction or hypersensitivity to previous vaccinations or vaccine component; fever at enrolment or acute infection up to 7 days before enrolment; any disorder associated with prolonged bleeding; immunosuppressive treatment within 30 days before enrolment; receipt of blood or blood products in the 12 weeks before enrolment; behavioural or cognitive impairment; and progressive or severe neurological disorder, seizure disorder, epilepsy, or Guillain-Barré syndrome. Twin pregnancies were included in the study (appendix). Carriage of GBS was not assessed or deemed an exclusion criterion for this study. Treatment of mothers and their babies, including HIV management, was provided by government services according to national guidelines, free at the point of care.

Gestational age was estimated by (in order of preference) ultrasonography before 24 weeks' gestation, date of last menstrual period, fundal height at 20–35 weeks' gestation, or ultrasonography at 25–27 weeks' gestation. CD4 cell counts were assessed using BD FACSCount (BD Biosciences, San Jose, CA, USA) and viral load was measured in Malawi using the Abbott RealTime HIV-1 assay (Abbott Laboratories, Abbott Park, IL, USA), and in South Africa using the Taqman version 2 HIV-1 assay (Roche, Pleasanton, CA, USA).

### Procedures

Women were immunised with a 0·5 mL dose of non-adjuvanted CRM_197_-conjugated GBS vaccine (Novartis Vaccines, Siena, Italy) containing 5 μg of each capsular polysaccharide of serotypes Ia, Ib, and III, reconstituted in 0·9% sodium chloride. The vaccine was given intramuscularly into the non-dominant arm at 24–35 weeks' gestation. For immunogenicity analysis, blood was collected from women before vaccination on day 1, at days 15 and 31 post-vaccination, and at delivery. For women infected with HIV, HIV-1 viral load and CD4 cell counts were measured before vaccination and the day of delivery. Cord blood or peripheral blood was collected from infants at birth and day 42.

GBS serotype-specific antibody concentrations in women and infants were estimated using a previously described ELISA protocol at GlaxoSmithKline Clinical Sciences Laboratory, Marburg, Germany.^18^ In brief, antibody concentrations were assessed using 96-well plates, which were coated in 1 μg/mL human serum albumin-conjugated GBS polysaccharide representing the three vaccine serotypes. Serially diluted serum samples were incubated on the coated plates for 1 h at 37°C and an alkaline-phosphatase-conjugated goat anti-human IgG secondary antibody was added after washing and incubated for a further 90 min. After further washing, SeramunGelb pNPP was added to the plates and incubated for 30 min at room temperature, then the reaction was stopped with SeramunGelb stop. Antibody concentrations were calculated using Mikrowin 2000 software from optical density values measured at 405 nm using a BEP III ELISA processor. Because of lab constraints, serotype Ia ELISA testing was done in three batches, whereas all the testing for serotype Ib and III was done in one batch. Antibody concentrations were expressed as geometric mean concentrations (GMCs) with 95% CIs, calculated with the Clopper-Pearson method. Subgroup analysis was done for women on the basis of baseline serotype-specific antibody titres.

### Outcomes

The primary objective of this study was to compare the amount of placental transfer of GBS serotype-specific antibodies to the infants of pregnant women infected with HIV and those not infected after administration of investigational GBS vaccine. As a secondary objective, the concentrations of maternal serotype-specific GBS antibodies were assessed post-vaccination. An exploratory objective was the assessment of the kinetics of maternally derived antibodies in infants born to vaccinated women. Safety objectives were the assessment of solicited adverse reactions, unsolicited adverse events, serious adverse events, and obstetric outcomes.

After immunisation, the women were observed for 30 min for any immediate adverse reactions. Adverse events and serious adverse events were graded on severity and possible relation to study vaccine by the investigator. A standardised approach was used to identify adverse events, which were defined as any untoward medical occurrence in a mother given the investigational vaccine or her infant that did not necessarily have a causal relation with this treatment. Solicited adverse reactions were recorded for 7 days post-vaccination with diary cards. Adverse events were collected up to day 31 post-vaccination, and adverse events requiring a physician's visit, serious adverse events, and deaths were recorded for women and infants throughout the study. As part of the safety analysis the outcome of the pregnancy was assessed, including the health status of neonates (including Apgar score). Admission to hospital for a normal delivery was not deemed a serious adverse event in the context of this study.

### Statistical analysis

The sample sizes were calculated on the conservative assumption that only 60 participants per group would be enrolled, that there would be a 15% dropout rate, and that 80% of women would deliver infants that are at 37 weeks or more of gestation or weighed 2500 g or more at birth. Thus, a sample size of 40 per group would give about 95% power to detect an 8% difference in the percentage of placental transfer between any two study groups. However, if the maximum number of participants per group (90) were to be reached, under the same assumptions for the dropout rate and for the percentage of babies born at 37 weeks or more of gestation or weighing 2500 g or more at birth, the number of assessable participants would increase up to 60 per group, giving about 95% power to detect a 6·5% difference in the percentage of placental transfer between any two study groups. After determination of the sample size for the trial with and without babies born at less than 37 weeks' gestation or less than 2500 g, we decided that both of these early gestation or underweight groups should be retained within the analysis. All serum analyses were done on the full analysis set, which included all mothers who correctly received the vaccine and who provided at least one valid assessable serum sample, and their neonates. Safety data were analysed descriptively for the safety set, which included all mothers who correctly received the study vaccination and who provided safety data, and their neonates.

The primary objective was analysed using an ANCOVA model on log_10_-transformed maternal and infant GBS antibody levels, with HIV group and country as qualitative factors, and gestational age at delivery and birthweight as covariates. Multiplicity of testing across the three participant groups was adjusted for by using a significance level of 0·016 (ie, p=0·05/3). The null hypothesis was rejected if a significant difference was seen for all three serotypes. The secondary objective was analysed using an ANCOVA on log_10_-transformed maternal antibody concentrations at each relevant timepoint, with HIV group and country as qualitative factors, and baseline log_10_-antibody concentration as a covariate. Infant antibody concentrations at each timepoint were analysed using an ANOVA model, with maternal HIV group and country as qualitative factors and a significance level of 0·05.

For analysis, any antibody concentrations that were below the lower limit of quantification (<LLQ) were set as half the LLQ (LLQ serotype Ia: 0·326 μg/mL, serotype Ib: 0·083 μg/mL, serotype III: 0·080 μg/mL). All statistical analyses were done with SAS version 9.1.

This study is registered with ClinicalTrials.gov, number NCT01412801.

### Role of funding source

The study sponsor was involved in all stages of the study, including manuscript development. The corresponding author had full access to all the data in the study and had final responsibility for the decision to submit for publication. All authors agreed to submit the manuscript for publication. No honorarium, grant, or other form of payment was provided to authors, with the exception of funding needed to do the study.

## Results

Of 398 women screened across two sites in Malawi and South Africa, 270 women and their infants were enrolled in the study between Sept 26, 2011, and Dec 4, 2012: 90 without HIV (45 from each country), 89 with HIV and high CD4 cell counts (44 from Malawi and 45 from South Africa), and 91 with HIV and low CD4 cell counts (46 from Malawi and 45 from South Africa; figure). 254 (94%) women completed the study across the three groups; seven were lost to follow-up, six withdrew consent, one died, and two relocated away from the study area. 256 (96%) of 266 infants enrolled completed the study across the groups. 270 mothers were enrolled in the study but only 260 remained in the study at delivery, five of whom had twins, resulting in 265 livebirths. One mother died during labour and had a stillbirth that was included in the enrolled infants (n=266). Reasons for withdrawal were death or stillbirth (n=8), and loss to follow-up (n=2).

Baseline characteristics for women and infants were similar across the groups, although, consistent with the natural history of HIV acquisition in these populations, women in the HIV-uninfected group were younger than those in the HIV-infected groups (table 1). No notable differences in demographics existed between sites except that the maternal median body-mass index was higher in all groups in South Africa (26·8–29·3 kg/m^2^) compared with Malawi (22·8–24·7 kg/m^2^). As expected, viral loads were higher in mothers infected with HIV and low CD4 cell counts than they were in those with high CD4 cell counts. Because most women who were not already receiving antiretroviral treatment were given treatment during the study, viral load decreased between screening and delivery in both HIV-infected groups. Rates of premature birth for the HIV-uninfected group were similar to those reported for sub-Saharan Africa, and were higher in HIV-infected groups.^19^

Rates of women reporting at least one solicited adverse reaction were highest in the HIV-uninfected group (60 [67%] of 90 women), compared with the low CD4 cell count (39 [44%] of 88 women) and high CD4 cell count (52 [59%] of 88 women) groups. Most reactions were grade 2 or less, with 4% or less of participants reporting a severe reaction for any of the solicited adverse reactions (table 2). Local reactions were reported by 18–39% of women across the groups, and 40–59% reported systemic reactions. The most frequently reported local adverse reaction was injection site pain, with severe pain being reported by two (2%) of 87 women with HIV and low CD4 cell count group and four (4%) of 90 in the HIV-uninfected group (table 2). The most often reported systemic adverse reactions were fatigue and headache, which were reported most frequently by women without HIV and least by those with HIV and low CD4 cell counts (table 2).

The percentage of women reporting adverse events was similar across the three groups, with 74–78% reporting at least one adverse event (table 3). Of these, 7–23% were deemed to be at least possibly related to the study vaccine. Serious adverse events were reported by 28–32% of women, but none were deemed to be caused by the study vaccine. Similarly, no differences in reporting of adverse events were seen across the infant groups. 41–49% of infants reported adverse events and 18–19% reported serious adverse events (table 3), none of which was deemed to be caused by vaccination. One woman with HIV in the high CD4 cell count group died from uterine rupture and her infant was stillborn (appendix). Seven other infant deaths occurred in the study, mostly in Malawi; no deaths in the study were deemed to be caused by the study vaccine (table 3, appendix).

No differences in obstetric outcomes and pregnancy events were recorded across the three groups (appendix). No association between vaccine administration and change in viral load was seen in the HIV-infected groups.

For all groups, GMCs of antibodies were higher post-vaccine than at baseline at all tested timepoints (table 4). The highest responses were seen in the HIV-uninfected group, for which ratios between baseline and delivery against the three serotypes were 15·0 μg/mL (Ia), 9·14 μg/mL (Ib), and 30 μg/mL (III); whereas these were 7·21 μg/mL (Ia), 4·83 μg/mL (Ib), and 9·99 μg/mL (III) for the low CD4 cell count group; and 8·78 μg/mL (Ia), 7·34 μg/mL (Ib), and 8·65 μg/mL (III) for the high CD4 cell count group. The biggest differences between women infected and not infected with HIV were reported against serotype III. No difference in immunogenicity was reported between mothers in the high and low CD4 cell count groups. Despite differences in magnitude, the kinetics of the antibody response in women infected and not infected with HIV (table 4), and the variation in individual responses were similar across groups (appendix). Antibody concentrations at baseline were undetectable (<LLQ) for about 69–80% of women against serotype Ia (72 in the low CD4 cell count, 66 in high CD4 cell count, and 62 in HIV-uninfected groups), 1–6% of women against serotype Ib (one in the low CD4 cell count, one in high CD4 cell count, and five in HIV-uninfected groups), and 34–43% of women against serotype III (22 in the low CD4 cell count, 33 in high CD4 cell count, and 33 in HIV-uninfected groups). Because too few participants had antibody concentrations below the LLQ against serotype Ib, subgroup analysis was only done on serotypes Ia and III. When stratified by baseline antibody concentration (<LLQ or ≥LLQ), differences in antibody GMCs between the HIV-uninfected and HIV-infected groups against serotypes Ia and III were less pronounced for women with undetectable GBS antibody concentrations at baseline. Antibody GMCs post-vaccination were higher in those who had detectable antibody concentrations at baseline, with GMCs at delivery of 18–61 μg/mL across groups for serotype Ia and 1·8–8·8 μg/mL for serotype III, compared with GMCs of 1·0–1·8 μg/mL and 0·4–1·1 μg/mL for the same serotypes for women who had undetectable antibody concentrations at baseline (appendix). Ratios of antibody GMCs to baseline could not be accurately calculated for this analysis because of the high frequency of women below the LLQ at baseline. For serotype Ia, a site difference in response was recorded, with women in South Africa having much higher baseline and vaccine-induced antibody responses than the women in Malawi, across the HIV-infected and HIV-uninfected groups (appendix).

Placental GBS serotype-specific antibody transfer ratios were similar between HIV-uninfected and HIV-infected groups for each serotype (0·49–0·72; table 5). Infant antibody GMCs followed the same pattern as maternal antibody GMCs, with infants in the HIV-uninfected group having higher GMCs at birth and day 42 than those in HIV-infected groups (table 5). Rates of antibody decay from day 1 to day 42 were similar across the three groups, with geometric mean ratios over this period of 0·50–0·58 for serotype Ia, 0·28–0·56 for serotype Ib, and 0·31–0·40 for serotype III.

## Discussion

In this small phase 2 trial in high disease burden settings in Malawi and South Africa, we showed the trivalent GBS vaccine to be well tolerated by pregnant women irrespective of HIV status, and have not identified any concerning safety signals in these women or their infants. The vaccine was substantially more immunogenic in women not infected with HIV than it was in those with the infection, and generated antibody GMCs to serotypes Ib and III similar to those recorded previously in pregnant women given this vaccine (GMCs at delivery from a previous study^18^ in Belgium and Canada: 2·41 μg/mL for serotype Ib and 1·90 μg/mL for serotype III). However, antibody GMCs against serotype Ia in mothers not infected with HIV in the present study were substantially lower than those recorded for the same vaccine dose in a previous study^17^ of women not infected with HIV in South Africa (4·49 μg/mL for the HIV-uninfected group in the present study compared with five times higher values for the group receiving the equivalent vaccine dose in the previous South African study). Carriage was not measured in the present study, but previous reports suggest that although South Africa and Malawi have similar prevalence in carriage isolates for serotype Ib (6·7% [South Africa] and 6·2% [Malawi]) and III (37% [South Africa] and 39% [Malawi]), they do have different prevalence for serotype Ia (30% [South Africa] and 18% [Malawi]).^20^, ^21^ In our study, of women not infected with HIV, ten (22%) of 45 in Malawi and 18 (40%) of 45 in South Africa had antibody GMCs more than or equal to the LLQ against serotype Ia at baseline, whereas in the low and high CD4 cell count groups, the numbers of women with GMCs in these ranges were two (4%) of 45 versus one (2%) of 44 in Malawi, and 16 (36%) of 45 versus 22 (49%) of 45 in South Africa, which might be due to this difference in carriage. However, the difference between these countries for serotype Ia should be interpreted with caution because of the small numbers of women in each group. We saw no similar patterns for the other two serotypes in our study. Although these studies had no major methodological differences between the assays used, these data emphasise the need to have an internationally standardised assay for the assessment of the response to GBS conjugate vaccines.

In Malawi and South Africa, 18–30% of pregnant women are infected with HIV and their infants are exposed to HIV.^22^, ^23^ Since HIV infection has been shown to reduce vaccine efficacy for a number of vaccines irrespective of CD4 cell counts,^16^ the lower immune response seen in the HIV positive groups compared with the uninfected group is unsurprising. This impaired response in individuals infected with HIV has also been seen for other conjugate vaccines.^24^

Antibody transfer ratios from mother to infant were similar across the HIV-infected and HIV-uninfected groups, and were similar to those ratios reported in previous trials^25^, ^26^ of this vaccine, and for other glycoconjugate vaccines given to pregnant mothers. A previous candidate conjugate GBS vaccine against serotype III led to antibody transfer rates of 77%, which was slightly higher than those seen in our study.^27^ Similarly, rates of antibody decay in infants were similar in our study to those seen in previous studies with candidate GBS vaccines; however, whether the antibody concentrations seen by day 42 would be sufficient to potentially protect against late-onset invasive GBS disease is not known. Baker and colleagues^27^ reported infant GBS serotype III-specific antibody concentrations of 30% of those recorded at birth at 2 months of age, and previous studies^18^ with the study vaccine reported infant concentrations of 22–32% of birth levels at 3 months of age. Although the functionality of maternal antibodies was not measured in our study, previous research has shown that functional GBS antibodies can persist for up to 2 years post-immunisation.^28^ Future vaccine studies would need to assess opsonophagocytic activity in the infant, in conjunction with an established correlate of protection, to estimate potential infant protection. Additionally, further research should concentrate on the responses of women who had undetectable antibody concentrations at baseline and vaccine strategies that might be needed to potentially provide protection to infants born to these women.

The assessment of GBS vaccines as they reach late-phase development would be greatly enhanced if the concentration of maternal and neonatal GBS antibody that correlates with passive protection against neonatal disease could be defined. Assay-specific antibody concentrations have been adopted to predict protection for conjugate vaccines against *H influenzae* type b and *S pneumoniae*, but although several thresholds for anti-GBS antibody have been proposed on the basis of naturally acquired and vaccine-acquired immunity,^29^, ^30^, ^31^ these thresholds have not been widely accepted, particularly in an African setting, and the absence of a standardised assay across studies makes the selection of a reliable correlate of protection difficult.^32^ A limitation of the immunogenicity testing in the present study is that the samples were analysed over a long period of time and multiple antigen lots, which could have contributed to the variation recorded. Several laboratories are working to generate a robust and widely applicable assay for approval by the regulatory authorities. Future larger scale studies should therefore focus on the use of a validated assay so that immunogenicity parameters can be readily compared between studies, countries, and over time. Our study is also limited by the small number of participants and the variation in baseline antibody concentrations, which led to differing magnitudes of individual responses to vaccination. Additional, larger studies are needed that include clinically relevant endpoints such as carriage, invasive disease, and mortality, in the appropriate populations. Future research should also include analysis of the effect of a booster vaccine dose, or a higher dose, on responses in pregnant women infected with HIV, or women with undetectable antibody concentrations at baseline.

In conclusion, the investigational glycoconjugate GBS vaccine was less immunogenic in women infected with HIV than those not infected, irrespective of CD4 cell count. The lower amounts of serotype-specific maternal antibody transferred to the infants of women infected with HIV compared with those not infected could reduce vaccine protection from neonatal GBS. At present, this vaccine is undergoing further phase 2 trials and studies of antibody persistence in pregnant women. Once a validated, approved assay and a correlate of protection has been established, researchers will be better able to understand the potential protective value of this vaccine for pregnant women and their infants in high HIV seroprevalence countries.

## Figures and Tables

**Figure:**
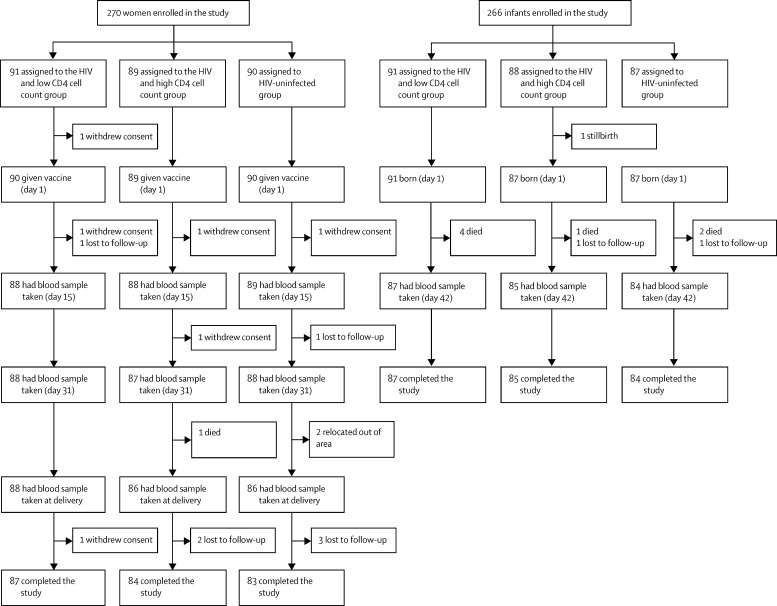
Study profile Most participants who did not complete the study were from the Malawi site, with the exception of the two women who relocated: one woman in the high CD4 cell count group who was lost to follow-up after delivery and one infant in the high CD4 cell count group who died between birth and day 42.

**Table 1:** Demographics and baseline characteristics of enrolled participants

	**HIV-infected, low CD4 cell count**	**HIV-infected, high CD4 cell count**	**HIV-uninfected**
**Women**			
n	91	89	90
Age (years)	28·0 (18–39)	28·0 (18–38)	24·0 (18–39)
Black, n (%)	91 (100%)	89 (100%)	90 (100%)
Body-mass index (kg/m^2^)	26·0 (17·9–48·7)	24·8 (16·0–43·5)	25·8 (20·3–43·4)
Time from vaccination to delivery (weeks)	11 (1–19)	10 (0–18)	9 (1–17)
Gestational age at vaccination (weeks)	27·0 (22–35)	29·0 (23–35)	29·5 (24–34)
CD4 cell count at baseline (cells per μL)	233 (55–348); n=89	478 (352–1099)	NA
CD4 cell count at delivery (cells per μL)	262 (30–988); n=81	502 (30–1326); n=80	NA
Viral load at baseline (RNA copies per mL)	2760 (20–814 356); n=90	305 (20–334 981); n=88	NA
Viral load at delivery (RNA copies per mL)	75 (20–240 965); n=80	75 (20–729 085); n=81	NA
Baseline HAART, n (%)	51 (56%)	47 (53%)	NA
**Infants**			
n	91	88	87
Male sex, n (%)	49 (54%)	48 (55%)	46 (53%)
Gestational age at birth (weeks)	39 (27–42)	38 (29–44)	39 (29–42)
Premature (<37 weeks), n (%)	19 (21%)	25 (28%)	14 (16%)
Early premature* (<32 weeks), n (%)	3 (3%)	2 (2%)	1 (1%)
Low birthweight (≤2·5 kg), n (%)	12 (13%)*	7 (8%)*	9 (10%)

Data are presented as the median and range unless stated otherwise and are from the full analysis set. NA=not applicable. HAART=highly active antiretroviral treatment.

* Two (2%) infants in each group were of very low birthweight (≤1·5 kg).

**Table 2:** Number of women reporting local and systemic adverse reactions during the first 7 days after vaccination

		**HIV-infected, low CD4 cell count (n=87)**	**HIV-infected, high CD4 cell count (n=88)**	**HIV-uninfected (n=90)**
**Local**				
Any local		16 (18%)	26 (30%)	35 (39%)
Ecchymosis		0	0	1 (1%)
Erythema		0	1 (1%)	1 (1%)*;* n=89
Induration		0	1 (1%)	0
Swelling		0	0	2 (2%)
Pain				
	Any	16 (18%)	26 (30%)	35 (39%)
	Severe	2 (2%)	0	4 (4%)
**Systemic**				
Any systemic		35 (40%)	48 (55%)	53 (59%)
Chills				
	Any	8 (9%)	12 (14%)	20 (22%)
	Severe	1 (1%)	1 (1%)	0
Nausea				
	Any	11 (13%)	15 (17%)	20 (22%); n=89
	Severe	1 (1%)	0	2 (2%); n=89
Malaise				
	Any	9 (10%)	17 (19%)	21 (23%)
	Severe	1 (1%)	0	1 (1%)
Myalgia		7 (8%)	15 (17%)	21 (23%)
Arthralgia				
	Any	11 (13%)	20 (23%)	26 (29%)
	Severe	0	0	1 (1%)
Headache				
	Any	21 (24%)	28 (32%)	39 (43%)
	Severe	1 (1%)	1 (1%)	2 (2%)
Fatigue				
	Any	21 (24%)	27 (31%)	42 (47%)
	Severe	2 (2%)	1 (1%)	3 (3%)
Rash		3 (3%)	1 (1%)	1 (1%)
Fever (≥38°C)		3 (3%)	0	0

For local adverse reactions, no participants reported severe reactions (>100 mm), with the exception of pain. Data are given for local reactions (≥25 mm).

**Table 3:** Number of women and infants reporting unsolicited adverse events and serious adverse events

	**HIV-infected, low CD4 cell count (90 women and 91 infants)**	**HIV-infected, high CD4 cell count (89 women and 88 infants)**	**HIV-uninfected (90 women and 87 infants)**
**Women**			
Any adverse event	67 (74%)	68 (76%)	70 (78%)
Adverse event possibly related to vaccine	6 (7%)	12 (13%)	21 (23%)
Serious adverse event*	25 (28%)	28 (31%)	29 (32%)
Adverse event leading to participant withdrawing from the trial	0	1 (1%)	0
Medically attended adverse event	42 (47%)	44 (49%)	49 (54%)
Death†	0	1 (1%)	0
**Infants**			
Any adverse event	37 (41%)	43 (49%)	37 (43%)
Adverse event possibly related to vaccine	0	2 (2%)	1 (1%)
Serious adverse event*	17 (19%)	16 (18%)	16 (18%)
Adverse event leading to participant withdrawing from the trial	4 (4%)	2 (2%)	2 (2%)
Medically attended adverse event	21 (23%)	29 (33%)	21 (24%)
Death†	4 (4%)	2 (2%)	2 (2%)

* No serious adverse events were deemed to be at least possibly related to vaccination.

† See appendix for details of deaths.

**Table 4:** Maternal geometric mean antibody concentrations and ratios to baseline

		**HIV-infected, low CD4 cell count**	**HIV-infected, high CD4 cell count**	**HIV-uninfected**	**Low CD4***vs***high CD4**	**Low CD4***vs***HIV-uninfected**	**High CD4***vs***HIV-uninfected**
**Serotype Ia**							
Data available, n		74	75	77	..	..	..
Geometric mean antibody concentration (μg/mL)							
	Day 1 pre-vaccination	0·24 (0·19–0·30)	0·25 (0·20–0·32)	0·38 (0·30–0·48)	p=0·709	p=0·007	p=0·019
	Day 15 post-vaccination	2·62 (1·64–4·17)	2·95 (1·86–4·69)	5·61 (3·56–8·84)	p=0·713	p=0·023	p=0·053
	Day 31 post-vaccination	2·68 (1·74–4·10)	3·26 (2·14–4·98)	6·63 (4·37–10)	p=0·513	p=0·003	p=0·020
	Delivery	2·22 (1·50–3·29)	2·69 (1·82–3·98)	4·49 (3·06–6·60)	p=0·493	p=0·013	p=0·067
Geometric mean ratio to day 1							
	Day 15	8·36 (5·27–13)	9·54 (6·02–15)	19 (12–30)	p=0·690	p=0·011	p=0·032
	Day 31	8·55 (5·60–13)	11 (6·91–16)	23 (15–35)	p=0·492	p=0·001	p=0·010
	Delivery	7·21 (4·88–11)	8·78 (5·95–13)	15 (10–22)	p=0·479	p=0·007	p=0·046
**Serotype Ib**							
Data available, n		44	53	66	..	..	..
Geometric mean antibody concentration (μg/mL)							
	Day 1 pre-vaccination	0·51 (0·38–0·70)	0·36 (0·27–0·48)	0·40 (0·31–0·51)	p=0·104	p=0·205	p=0·652
	Day 15 post-vaccination	2·93 (1·73–4·95)	3·50 (2·17–5·65)	6·07 (3·98–9·26)	p=0·617	p=0·035	p=0·090
	Day 31 post-vaccination	2·62 (1·62–4·24)	3·68 (2·38–5·70)	5·35 (3·63–7·87)	p=0·300	p=0·024	p=0·209
	Delivery	2·12 (1·36–3·31)	3·04 (2·03–4·56)	3·84 (2·69–5·50)	p=0·236	p=0·042	p=0·392
Geometric mean ratio to day 1							
	Day 15	6·87 (4·08–12)	8·23 (5·12–13)	14 (9·36–22)	p=0·611	p=0·033	p=0·089
	Day 31	6·11 (3·79–9·85)	8·70 (5·63–13)	13 (8·56–19)	p=0·277	p=0·021	p=0·211
	Delivery	4·83 (3·10–7·52)	7·34 (4·90–11)	9·14 (6·38–13)	p=0·165	p=0·029	p=0·425
**Serotype III**							
Data available, n		53	55	70	..	..	..
Geometric mean antibody concentration (μg/mL)							
	Day 1 pre-vaccination	0·12 (0·09–0·17)	0·10 (0·07–0·14)	0·14 (0·10–0·18)	p=0·407	p=0·572	p=0·145
	Day 15 post-vaccination	1·24 (0·79–1·95)	1·52 (0·97–2·36)	5·90 (3·99–8·72)	p=0·528	p<0·0001	p<0·0001
	Day 31 post-vaccination	1·51 (0·97–2·35)	1·31 (0·85–2·02)	5·35 (3·66–7·83)	p=0·643	p<0·0001	p<0·0001
	Delivery	1·25 (0·81–1·94)	1·07 (0·70–1·65)	3·80 (2·61–5·55)	p=0·616	p=0·0002	p<0·0001
Geometric mean ratio to day 1							
	Day 15	9·85 (6·25–16)	12 (7·64–18)	47 (32–70)	p=0·555	p<0·0001	p<0·0001
	Day 31	12 (7·70–19)	10 (6·62–16)	43 (29–63)	p=0·599	p<0·0001	p<0·0001
	Delivery	9·99 (6·44–15)	8·65 (5·65–13)	30 (21–44)	p=0·638	p=0·0002	p<0·0001

Data are geometric means (95% CI), unless otherwise stated. p values for pairwise comparisons are given in the last three columns (ANCOVA).

**Table 5:** Infant antibody GMCs, GMRs, and mean placental transfer ratios

	**HIV-infected, low CD4 cell count**		**HIV-infected, high CD4 cell count**		**HIV-uninfected**		**Low CD4***vs***high CD4**	**Low CD4***vs***HIV-uninfected**	**High CD4***vs***HIV-uninfected**
	n	Data	n	Data	n	Data			
**Serotype Ia**									
Transfer ratio*	79	0·58 (0·49–0·69)	79	0·60 (0·51–0·72)	83	0·72 (0·61–0·85)	p=0·755	p=0·089	p=0·169
GMC day 1 (μg/mL)	79	1·01 (0·66–1·56)	81	1·22 (0·80–1·87)	83	3·91 (2·56–5·96)	p=0·548	p<0·0001	p=0·0002
GMC day 42 (μg/mL)	81	0·61 (0·41–0·89)	83	0·64 (0·44–0·94)	79	1·97 (1·33–2·91)	p=0·848	p<0·0001	p<0·0001
GMR day 42:day 1	70	0·58 (0·50–0·67)	78	0·50 (0·44–0·57)	75	0·50 (0·44–0·58)	p=0·296	p=0·344	p=0·925
**Serotype Ib**									
Transfer ratio*	41	0·51 (0·38–0·69)	54	0·64 (0·50–0·83)	57	0·49 (0·38–0·63)	p=0·251	p=0·796	p=0·131
GMC day 1 (μg/mL)	44	1·31 (0·78–2·19)	56	1·62 (1·03–2·56)	57	2·67 (1·70–4·20)	p=0·537	p=0·040	p=0·125
GMC day 42 (μg/mL)	75	0·29 (0·19–0·44)	70	0·44 (0·29–0·68)	67	1·16 (0·75–1·80)	p=0·174	p<0·0001	p=0·002
GMR day 42:day 1	34	0·28 (0·20–0·38)	46	0·35 (0·27–0·46)	46	0·56 (0·42–0·73)	p=0·297	p=0·0006	p=0·008
**Serotype III**									
Transfer ratio*	53	0·60 (0·44–0·82)	44	0·51 (0·36–0·72)	66	0·56 (0·43–0·75)	p=0·515	p=0·791	p=0·670
GMC day 1 (μg/mL)	54	0·60 (0·36–0·99)	51	0·52 (0·31–0·88)	66	3·88 (2·47–6·10)	p=0·713	p<0·0001	p<0·0001
GMC day 42 (μg/mL)	77	0·21 (0·14–0·31)	80	0·15 (0·10–0·22)	77	0·86 (0·58–1·28)	p=0·258	p<0·0001	p<0·0001
GMR day 42:day 1	43	0·39 (0·32–0·49)	46	0·40 (0·33–0·49)	59	0·31 (0·26–0·38)	p=0·574	p=0·186	p=0·051

95% CIs are given in parenthesis. p values for pairwise comparisons are given in the last three columns (ANOVA for transfer ratio and ANCOVA for geometric mean antibody concentrations and GMRs). GMC=geometric mean antibody concentrations. GMR=ratio of GMCs to baseline.

* Transfer ratio=infant GMC in blood collected within 72 h of birth divided by maternal GMC in blood collected at delivery.
