# Supplementary material for: Group B streptococcus vaccination in pregnant women with or without HIV in Africa: a non-randomised phase 2, open-label, multicentre trial
Source: Lancet Infect Dis. 2016 May;16(5):546–55. doi: 10.1016/S1473-3099(15)00484-3 (PMC4835545; doi:10.1016/S1473-3099(15)00484-3)

THE LANCET Infectious Diseases

Supplementary webappendix

This webappendix formed part of the original submission and has been peer reviewed. We post it as supplied by the authors.

Supplement to: Heyderman RS, Madhi SA, French N, et al. Group B streptococcus vaccination in pregnant women with or without HIV in Africa: a non-randomised phase 2, open-label, multicentre trial. *Lancet Infect Dis* 2016; published online Feb 8. http://dx.doi.org/10.1016/S1473-3099(15)00484-3.

Supplementary Table 1: Causes of maternal and infant deaths

Vaccine Group	Cause of death	Age at death (days)	Country	Relatedness to study vaccine
Women				
HIV-infected CD4High	▪ Disseminated intravascular coagulation<br>▪ Hemorrhagic shock<br>▪ Uterine rupture	Study Day 72	Malawi	None
Infants				
HIV-uninfected	▪ Neonatal asphyxia	1	Malawi	None
HIV-uninfected	▪ Hypoxic-ischemic encephalopathy<br>▪ Neonatal asphyxia<br>▪ Neonatal aspiration<br>▪ Neonatal sepsis	1	Malawi	None
HIV-infected CD4Low	▪ Chromosomal deletion<br>▪ Neonatal asphyxia<br>▪ Neonatal aspiration	1	Malawi	None
HIV-infected CD4Low	▪ Neonatal sepsis	14	Malawi	None
HIV-infected CD4Low	▪ Neonatal respiratory distress syndrome<br>▪ Premature baby	1	Malawi	None
HIV-infected CD4Low	▪ Neonatal respiratory distress syndrome<br>▪ Premature baby<br>▪ Petechiae	1	Malawi	None
HIV-infected CD4High	▪ Stillbirth	1	Malawi	None
HIV-infected CD4High	▪ Gastroenteritis<br>▪ Hypernatremia<br>▪ Metabolic acidosis<br>▪ Renal impairment<br>▪ Shock	4	S. Africa	None

Supplementary Table 2: Number and percentage of women reporting pregnancy events and obstetric outcomes, by HIV status group

	**HIV-infected CD4Low** *n=90*	**HIV-infected CD4High** *n=89*	**HIV-uninfected** *n=90*
Pregnancy events			
Chorioamnionitis	1 (1%)	0	0
Cephalopelvic disproportion	0	0	1 (1%)
(Pre-) eclampsia	0	1 (1%)	1 (1%)
Failed induction	1 (1%)	2 (2%)	0
Fetal distress	1 (1%)	0	0
Gestational diabetes	0	1 (1%)	0
Lacerations/episiotomy	24 (27%)	28 (31%)	28 (31%)
Premature rupture of membranes	0	1 (1%)	0
Prolonged labour	0	1 (1%)	0
Retained placenta and post-partum haemorrhage	0	1 (1%)	0
Delivery Type			
Caesarean	20 (22%)	15 (17%)	16 (18%)
Vaginal	62 (69%)	68 (76%)	66 (73%)
Vacuum	1 (1%)	0	1 (1%)
Forceps or ventouse	0	0	2 (2%)
Not reported	7 (8%)	6 (7%)	5 (6%)
Infants			
	n=91	*n=88*	*n=87*
Live birth singletons	85 (93%)	85 (97%)	85 (98%)
Live birth twins	6 (7%)	2 (2%)	2 (2%)
Stillbirth	0	1 (1%)	0

Supplementary Table 3: Geometric mean GBS serotype Ia and III-specific antibody concentrations (GMCs) for HIV positive and HIV negative women, separated by baseline antibody status, relative to lower limit of quantification (LLQ). GMCs are expressed as μg/mL and 95% confidence intervals are given in parenthesis.

	**Baseline < LLQ**			**Baseline ≥ LLQ**		
	**HIV-infected CD4Low**	**HIV-infected CD4High**	**HIV-uninfected**	**HIV-infected CD4Low**	**HIV-infected CD4High**	**HIV-uninfected**
Serotype Ia						
Day 1 pre-vaccination	0.16 (0.16–0.16) *n=72*	0.16 (0.16–0.16) *n=66*	0.16 (0.16–0.16) *n=62*	0.98 (0.58–1.65) *n=18*	0.67 (0.41–1.11) *n=23*	1.81 (1.24–2.64) *n=28*
Day 15 post-vaccination	1.03 (0.63–1.69) *n=65*	1.29 (0.77–2.15) *n=61*	1.83 (1.09–3.08) *n=59*	41 (19–90) *n=17*	20 (9.63–41) *n=23*	105 (61–180) *n=28*
Day 31 post-vaccination	1.04 (0.63–1.69) *n=66*	1.40 (0.85–2.32) *n=57*	2.30 (1.39–3.81) *n=56*	41 (21–80) *n=17*	22 (12–41) *n=23*	105 (66–166) *n=28*
Delivery	1.0 (0.66–1.51) *n=68*	1.11 (0.71–1.75) *n=58*	1.77 (1.12–2.79) *n=56*	35 (16–77) *n=15*	18 (9–34) *n=23*	61 (37–101) *n=27*
Serotype III						
Day 1 pre-vaccination	0.04 (0.04–0.04) *n=22*	0.04 (0.04–0.04) *n=33*	0.04 (0.04–0.04) *n=33*	0.22 (0.17–0.30) *n=43*	0.25 (0.19–0.33) *n=44*	0.28 (0.22–0.37) *n=49*
Day 15 post-vaccination	0.4 (0.19–0.82) *n=21*	0.4 (0.22–0.74) *n=30*	1.66 (0.92–3.02) *n=31*	2.46 (1.23–4.89) *n=39*	2.96 (1.49–5.88) *n=37*	16 (8.7–29) *n=48*
Day 31 post-vaccination	0.45 (0.22–0.90) *n=21*	0.39 (0.21–0.70) *n=30*	1.59 (0.89–2.86) *n=30*	3.29 (1.66–6.49) *n=38*	1.97 (1.01–3.83) *n=37*	14 (7.53–25) *n=46*
Delivery	0.41 (0.22–0.76) *n=21*	0.39 (0.23–0.65) *n=32*	1.12 (0.66–1.90) *n=29*	1.87 (0.98–3.55) *n=40*	1.79 (0.88–3.64) *n=31*	8.84 (4.93–16) *n=46*

Note: analysis not performed for serotype Ib as too few women had a baseline GMC < LLQ

Supplementary Table 4: Geometric mean antibody concentrations (95% confidence intervals) by HIV status and site.

	**HIV-infectedCD4Low**		**HIV-infectedCD4High**		**HIV-uninfected**	
	**S. Africa**	**Malawi**	**S. Africa**	**Malawi**	**S. Africa**	**Malawi**
**Serotype Ia**						
Day 1 (pre-vaccination)	0.34 (0.24–0.50) *n=45*	0.17 (0.15–0.20) *n=45*	0.37 (0.25–0.54) *n=45*	0.17 (0.14–0.20) *n=44*	0.48 (0.33–0.70) *n=45*	0.26 (0.22–0.31) *n=45*
Day 15 post-vaccination	5.14 (2.90–9.12) *n=45*	1.25 (0.65–2.40) *n=37*	6.13 (3.46–11.0) *n=45*	1.35 (0.71–2.56) *n=39*	10 (5.77–18) *n=45*	1.73 (0.92–3.26) *n=42*
Day 31 post-vaccination	4.60 (2.75–7.69) *n=45*	1.44 (0.78–2.66) *n=38*	6.63 (3.97–11.0) *n=45*	1.49 (0.79–2.82) *n=35*	11 (6.68–19) *n=45*	2.19 (1.17–4.11) *n=39*
Delivery	4.34 (2.66–7.09) *n=43*	1.05 (0.61–1.81) *n=40*	5.02 (3.11–8.11) *n=45*	1.09 (0.62–1.91) *n=36*	7.28 (4.43–12) *n=42*	1.74 (1.01–3.01) *n=41*
**Serotype Ib**						
Day 1 (pre-vaccination)	0.52 (0.37–0.74) *n=34*	0.46 (0.31–0.66) *n=29*	0.42 (0.30–0.59) *n=38*	0.31 (0.22–0.43) *n=32*	0.46 (0.33–0.65) *n=35*	0.37 (0.27–0.50) *n=42*
Day 15 post-vaccination	2.51 (1.41–4.48) *n=40*	2.39 (1.00–5.71) *n=30*	2.70 (1.57–4.66) *n=42*	3.45 (1.58–7.55) *n=34*	5.24 (3.04–9.02) *n=44*	7.45 (3.95–14) *n=40*
Day 31 post-vaccination	1.85 (1.11–3.06) *n=41*	1.70 (0.83–3.50) *n=34*	2.82 (1.73–4.60) *n=43*	2.82 (1.39–5.73) *n=34*	4.20 (2.55–6.90) *n=45*	5.86 (3.29–10) *n=39*
Delivery	1.80 (1.09–2.97) *n=36*	1.87 (0.95–3.70) *n=38*	2.68 (1.71–4.19) *n=43*	2.02 (1.06–3.86) *n=37*	2.97 (1.86–4.75) *n=42*	5.29 (3.06–9.15) *n=40*
**Serotype III**						
Day 1 (pre-vaccination)	0.17 (0.11–0.25) *n=40*	0.092 (0.061–0.14) *n=25*	0.11 (0.073–0.16) *n=40*	0.11 (0.082–0.16) *n=37*	0.16 (0.11–0.23) *n=44*	0.11 (0.076–0.15) *n=38*
Day 15 post-vaccination	1.25 (0.75–2.08) *n=42*	1.66 (0.74–3.71) *n=34*	1.93 (1.14–3.28) *n=41*	0.89 (0.45–1.73) *n=36*	8.77 (5.41–14) *n=45*	4.10 (2.21–7.63) *n=41*
Day 31 post-vaccination	1.35 (0.79–2.28) *n=42*	2.26 (1.07–4.78) *n=36*	1.34 (0.78–2.30) *n=42*	0.77 (0.41–1.44) *n=35*	6.58 (4.03–11) *n=45*	4.35 (2.38–7.95) *n=37*

Delivery	1.10 (0.68–1.77) *n=43*	1.09 (0.53–2.24) *n=40*	1.32 (0.80–2.20) *n=39*	0.72 (0.37–1.37) *n=33*	4.20 (2.63–6.73) *n=42*	2.92 (1.64–5.22) *n=40*

Supplementary Figure 1: Boxplots of individual maternal antibody concentrations against each serotype at baseline and delivery.

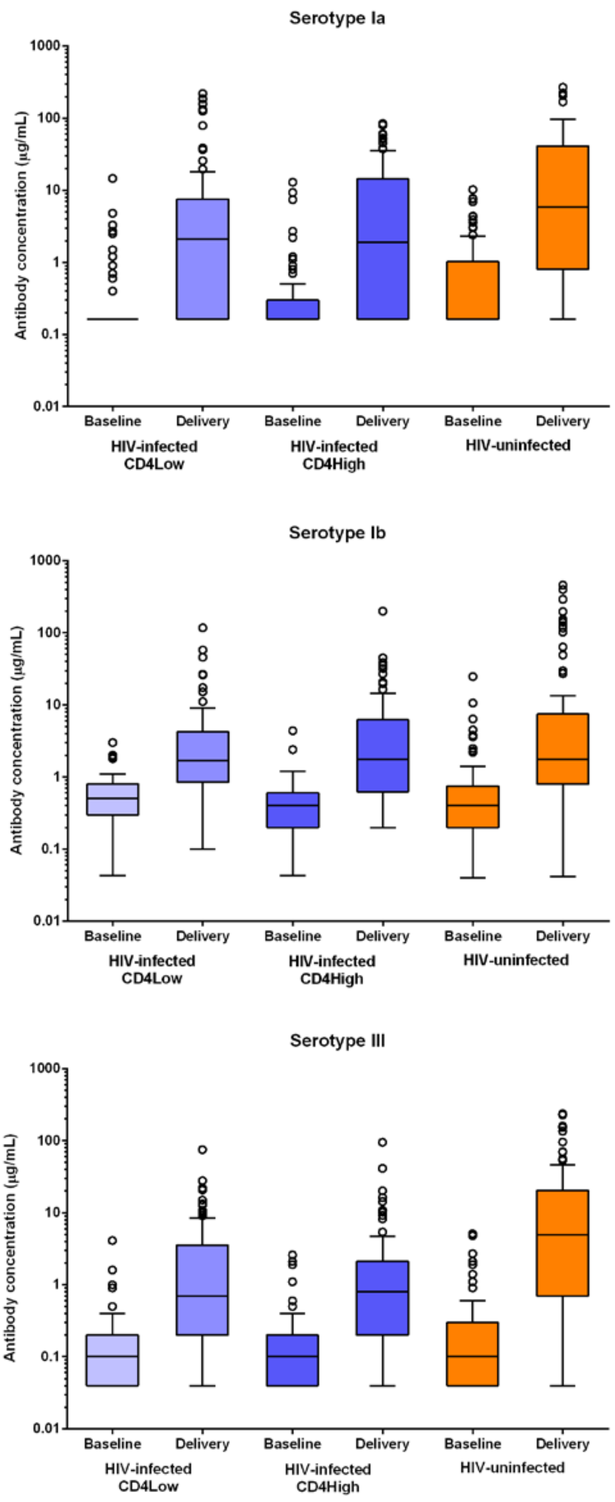

Supplement: Supplementary appendix [file mmc1.pdf]
